# Altered late semantic processing of food stimuli in female anorexia nervosa and atypical anorexia nervosa patients

**DOI:** 10.3389/fnhum.2026.1912696

**Published:** 2026-09-03

**Authors:** Panagiotis Loizou, Styliani Kaimakami, Angelina Tomazou, Georgia Panayiotou, Panos Zanos, Markus Junghöfer, Evangelos Paraskevopoulos

**Affiliations:** 1 Department of Psychology, University of Cyprus, Nicosia, Cyprus; 2 Center for Applied Neuroscience (CAN), Department of Psychology, University of Cyprus, Nicosia, Cyprus; 3 Institute for Biomagnetism and Biosignalanalysis, University of Münster, Münster, Germany; 4 Otto Creutzfeldt Center for Cognitive and Behavioral Neuroscience, University of Münster, Münster, Germany

**Keywords:** anorexia nervosa, atypical anorexia, food, EEG, semantic processing

## Abstract

**Introduction:**

Anorexia Nervosa (AN) patients exhibit cognitive and neural disturbances during food processing, yet underlying mechanisms remain unclear. Our study investigated whether AN and Atypical AN (AAN) patients show altered neural, cognitive, and behavioral responses to high and low-calorie food stimuli, and whether these effects appear largely food-specific.

**Methods:**

Seventeen AN/AAN, 17 HCs, and 17 overweight/obese females viewed high and low-calorie food stimuli, rated palatability (yes/no), and completed an emotional picture viewing task (IAPS) while high-density EEG was recorded.

**Results:**

Palatability ratings did not differ significantly among groups; however, exploratory analyses suggested that patients with the restricting subtype (AN-R; *n* = 6) rated high-calorie foods as less palatable than low-calorie foods compared to other groups. EEG findings revealed significant but modest group-specific modulation (N400; 388–418 ms; *p* = 0.049; Cluster size = 97 voxels; Peak statistic *X* = 9.78): HCs showed enhanced responses to high-calorie food, whereas AN/AAN and overweight/obese participants showed greater responses to low-calorie food. Source localization implicated the right fusiform gyrus and inferior-medial temporal cortices. No comparable effects occurred during the IAPS task.

**Conclusion:**

AN/AAN individuals exhibit disrupted calorie-dependent semantic processing in right inferior temporal regions, indicating altered food evaluation. These findings provide a neural basis for restrictive eating in AN/AAN, highlighting the role of semantic processing in disorder-specific food disturbances.

## Highlights

1) AN/AAN patients show altered semantic (N400) responses to food stimuli.
2) Altered food processing in AN/AAN is localized to the right inferior-temporal cortex.
3) The AN/AAN group exhibited a reversed ERP activation pattern during food stimulus processing.

## 1 Introduction

Fundamental clinical features in Anorexia Nervosa (AN) include intense fear of weight gain, disturbance in body shape or weight perception, and the maintenance of a low Body Mass Index (BMI) (American Psychiatric Association, 2013). Individuals who meet the first two criteria and engage in caloric restriction yet remain within or above the normal BMI may be assigned a diagnosis of Atypical Anorexia Nervosa (AAN) (Harrop et al., 2021). Both AN and AAN individuals share several clinical, psychological and behavioral characteristics (Moskowitz and Weiselberg, 2017), as well as overlapping metacognitive processes related to eating-disordered psychopathology (Davenport et al., 2015).

Food-related motivation in AN is increasingly conceptualized as a multifaceted construct encompassing cognitive, emotional, and reward-related processes (Godier et al., 2016; Joos et al., 2011; Paslakis et al., 2016; Scharner and Stengel, 2019). Evidence across behavioral, neuroimaging, and electrophysiological studies suggests that individuals with AN exhibit systematic alterations in these domains when processing food cues (Cowdrey et al., 2013; Uher et al., 2003; Frausto et al., 2022).

AN patients exhibit a decision-making pattern characterized by heightened perceived risk in food choices compared to Healthy Controls (HCs) (Lakritz et al., 2023). Reduced explicit and implicit “wanting” for High-Calorie Food (HCF) and greater implicit “wanting” for Low-Calorie Food (LCF) have been reported in both current and weight-restored AN patients compared to HCs (Cowdrey et al., 2013). Similarly, AN patients exhibited a diminished explicit motivational approach toward HCF compared to HCs, as reflected by reduced urge ratings (Paslakis et al., 2016). These findings suggest that both conscious and automatic motivational processes contribute to food-related avoidance in AN, supporting the notion of a global loss of incentive value of HCF. Moreover, when examining these biases, it is important to acknowledge that caloric density is confounded with food type and perceived healthiness. This was showcased in the study of Paslakis et al. Glasofer et al. (2016), where both HCs and AN individuals rated HCF (which were predominantly sweet/highly processed) as unhealthy, and LCF (which were predominantly fresh, unprocessed produce) as healthy.

Further evidence of the multifaceted food-related motivation in AN is reflected by the dissociation between subjective and neurophysiological measures. For instance, self-report palatability and craving ratings indicated reduced food engagement in current AN patients, while neuroimaging findings revealed enhanced neural responses to food compared to HCs, including heightened early magnetoencephalography (MEG) activity in the left occipito-temporal and inferior frontal regions in adolescent AN patients (Frausto et al., 2022). Likewise, explicit self-report measures in recovered AN patients were similar to HCs; however, functional magnetic resonance imaging (fMRI) findings showed increased medial prefrontal cortex and anterior cingulate activation compared to HCs (Uher et al., 2003). These findings indicate that altered neural processing in response to food is not fully captured by explicit self-report measures in AN (Frausto et al., 2022; Uher et al., 2003). This dissociation has motivated further investigations into how implicit neurophysiological measures capture aspects of food processing that are not accessible by subjective approaches (Loizou et al., 2024; Uher et al., 2003).

Further insights into AN patients’ food-related motivation emerge from their neural processing at different stages. During early processing (220–310 ms), a generalized attentional bias toward both HCF and LCF stimuli was observed in AN patients, whereas in HCs this bias appeared primarily toward HCF stimuli (Blechert et al., 2011a). Further evidence showed enhanced early processing of food-related stimuli (150 ms) in the acute AN group, but not in recovered AN patients or HCs (Godier et al., 2016). Such evidence suggests heightened preconscious/automatic attentional processing that potentially contributes to the maintenance of restrictive eating behavior (Godier et al., 2016). Beyond early attentional biases, later attentional biases indicate motivated attention and the subjective emotional salience of food stimuli in AN patients (Wolz et al., 2015). According to Novosel et al. (2014), AN patients exhibited enhanced Late Positive Potential (LPP) amplitude during exposure to food stimuli, particularly for LCF stimuli, an effect interpreted as reflecting heightened motivational and attentional relevance toward these stimuli. Converging evidence of heightened activation in medial prefrontal and anterior cingulate regions in recovered AN patients during food processing further supports alterations at later evaluative stages (Uher et al., 2003).

To date, numerous studies have shown that AN patients exhibit significant disturbances in neural circuits underlying emotion and reward processing, including the limbic system and reward-related areas during exposure to food-related cues (Ellison et al., 1998; Joos et al., 2011; Scharner and Stengel, 2019), or gustatory cues (Vocks et al., 2011). However, it remains unclear whether these alterations extend to more general affective stimuli. Only a limited number of studies have employed standardized emotional paradigms, such as the International Affective Picture System (IAPS), to address this question (Horndasch et al., 2018b; Nandrino et al., 2012). Subjective approaches found that despite group differences in neural activation, the valence ratings of IAPS pictures did not differ between adult AN patients and HCs (Horndasch et al., 2018b). Similarly, no significant differences were reported between AN patients and HCs in subjective valence or arousal ratings, suggesting the absence of a generalized impairment in emotion processing (Blechert et al., 2011a). In contrast, Nandrino et al. (2012) reported that AN patients rated positive IAPS pictures as more intense and made more errors for positive and neutral stimuli. Taken together, subjective findings remain inconclusive regarding whether emotional processing disturbances in AN generalize beyond food stimuli.

Neurophysiological evidence is similarly inconsistent. While no group differences were observed in early electroencephalography (EEG) responses to general emotional stimuli (Blechert et al., 2011a), fMRI findings indicated neurophysiological differences between the two groups (Horndasch et al., 2018b). Similarly, Mallorquí-Bagué et al. (2020) reported reduced P300 amplitudes in AN patients compared to HCs across both emotional (IAPS) and food-craving tasks, suggesting a potential general alteration in their neurophysiological responses. Such findings raise questions about whether the neurophysiological mechanisms underlying emotion and food-related processing in AN reflect a shared pathway, pointing toward a generalized disruption in emotional reactivity and salience processing.

Based on the above, the current study sought to elucidate the neural and behavioral differences in the processing of HCF and LCF stimuli among AN/AAN patients, OW/OB participants, and HCs. We hypothesized that AN patients, OW/OB participants, and HCs would differ in their behavioral responses to food stimuli, as reflected in palatability ratings, with AN patients expected to rate HCF as less palatable than the other groups. Furthermore, we hypothesized that AN/AAN, OW/OB, and HCs would show distinct neural responses to food stimuli as measured via high-density EEG analyzed in sensor-space. Finally, by assessing the processing of IAPS pictures, we also aimed to address whether the observed food stimuli alterations are largely food-specific or extend to general emotional processing.

## 2 Materials and methods

### 2.1 Research design

A cross-sectional quasi-experimental design was followed using a mixed-model approach. Concerning the methodological approach, a multimodal integration method comprising electrophysiological (EEG), behavioral (yes/no palatability questions), and self-report data (questionnaires) was used to assess the mechanisms employed during the processing of the presented stimuli. The factors in the model included the category of stimuli and the participants’ group.

### 2.2 Participants

An *a priori* power calculation performed using G*Power version 3.1.9.7 indicated that a minimum of 27 participants was required in total. The calculation was performed for an *F*-test, with an effect size *f* = 0.43, a significance criterion of *α* = 0.05, and a desired power of 0.95. The effect size was chosen based on the “category × participant group” interaction effect (*η^2^* = 0.16) reported in a similar study (Horndasch et al., 2018a). However, to ensure sufficient statistical power, 17 participants diagnosed with AN or AAN, 17 HCs, and 17 overweight/obese (OW/OB) volunteers without any Eating Disorders (EDs) were recruited, totaling 51 participants. The inclusion of the OW/OB control group, in addition to HCs, allowed us to disentangle neural and cognitive alterations that are specific to restrictive food intake from those that may be related to a linear relationship between body weight and brain functioning (Sun et al., 2024). Regarding inclusion criteria, all participants were required to have a good comprehension of the Greek language, defined as the ability to understand the study instructions, complete the Greek-language questionnaires, and follow the experimental procedures conducted in Greek language. No formal standardized language-comprehension test was administered. For the experimental group, participants had to meet the diagnostic criteria for AN or AAN based on the Eating Disorder Assessment-for DSM-5 (EDA-5) interview. Additionally, the Eating Attitudes Test-26 (EAT-26) scores were used to complement the EDA-5 findings. Regarding the control groups (HCs and OW/OB), participants were required to have a BMI within the ranges of 18.5–24.9 and ≥ 25 respectively, to meet no criteria for any EDs based on the EDA-5 interview and EAT-26 total score, and to have no history of any EDs. Exclusion criteria for all participants included color blindness, intellectual or neurological disabilities, alcohol or drug abuse, pervasive developmental disorders, visual impairments, epilepsy, and a history of bariatric surgery. Individuals in the control groups who reported having a current mental health diagnosis, had a history of EDs, screened positive for ED symptomatology on the EAT-26, or were identified as meeting criteria for an ED based on the EDA-5 assessment were excluded.

Specifically, during the screening process, two participants from the OW/OB group were excluded: one due to a previous diagnosis of binge-eating disorder and one due to a permanent partial visual impairment. Three individuals from the HC group were excluded in total: one due to a history of EDs (past diagnosis of AN) and two due to low BMI (underweight range). No participants were excluded due to other mental health conditions.

### 2.3 Procedure

Participants were instructed to consume a light meal (e.g., a snack) 3 hours prior to the experimental start and to avoid smoking, drinking alcohol, or caffeine-containing beverages, 4 hours prior to the experiment. Upon arrival at the lab (University of Cyprus), participants provided informed consent and completed a demographic questionnaire assessing their eligibility, followed by the completion of the Depression, Anxiety and Stress Scale-21 (DASS-21), EAT-26, and the Body Image-Acceptance and Action Questionnaire (BI-AAQ) via Google Forms. The participants were seated 60 cm from a computer monitor and wore an EEG electrode cap throughout the entire session. The experiment consisted of two blocks, each lasting approximately 6 minutes, with the full session lasting around 60 min. In the first block, 60 HCF and 60 LCF pictures were presented for 1,500 ms, each preceded by a 200 ms fixation cross, and followed by a yes/no palatability question (“Was the presented food palatable?”) that participants answered using a mouse button press. The second block involved the continuous presentation of 60 positive (high valence—low arousal) and 60 negative (low valence—high arousal) IAPS stimuli, presented for 1,500 ms, each preceded by a 200 ms fixation cross, without any behavioral questions. Stimulus presentation was carried out by Presentation® software (Version 23.0). Following the completion of the experiment, the EDA-5 interview was conducted by the first author, a fourth-year doctoral candidate in Clinical Psychology specializing in EDs who was trained in EDA-5 administration using the official EDA-5 user guide (Glasofer et al., 2016). As all EDA-5 interviews were conducted by a single interviewer, inter-rater reliability was not calculated in the present study. Clinician ratings and diagnostic judgments were handled according to the standardized EDA-5 assessment framework (Glasofer et al., 2016), by adhering to the EDA-5 digital interface’s sequential branching logic, standardized probe questions, and neutral clarifying prompts for ambiguous responses aiming to minimize interviewer bias and ensure consistent evaluation. To assess EDs symptomatology alongside the clinical interview, the EAT-26 was administered. Additionally, participants’ body weight was measured. Regarding dropouts, the experimental procedure was completed in a single lab visit, therefore, no dropouts occurred.

### 2.4 Measures

#### 2.4.1 Eating attitudes test-26 (EAT-26)

The Eating Attitudes Test-26 (EAT-26) is a widely used 26-item Likert-scale self-report questionnaire assessing eating disorder-related attitudes and behaviors. It comprises three subscales: Dieting (13 items), Bulimia and Food Preoccupation (6 items), and Oral Control (7 items) (Garfinkel, 1982; Garner et al., 1982). The EAT-26 has been adapted for use in the Greek population and has demonstrated good psychometric properties (Kosmidou et al., 2018; Venetsanou and Ioannidou, 2019).

#### 2.4.2 Body image–acceptance and action questionnaire (BI-AAQ)

The Body Image–Acceptance and Action Questionnaire (BI-AAQ) was utilized to provide additional information regarding our participants’ body image inflexibility. The BI-AAQ is a 12-item questionnaire using a seven-point Likert scale, with higher scores indicating greater body image inflexibility (Lee et al., 2018). The questionnaire has demonstrated excellent psychometric properties (Sandoz et al., 2013). The Greek version has shown a unifactorial structure, good internal consistency (*α* = 0.95), and satisfactory item-total correlations (Mavraki et al., 2020).

#### 2.4.3 Depression, anxiety and stress scale-21 (DASS-21)

The Depression, Anxiety and Stress Scale-21 (DASS-21) is a 21-item Likert-type questionnaire assessing symptoms of depression, anxiety, and stress (Lovibond, 1995). The Greek version has demonstrated satisfactory internal consistency (α = 0.85, 0.84, and 0.84), and validity for the three subscales (Pezirkianidis et al., 2018).

#### 2.4.4 Eating disorder assessment for DSM-5 (EDA-5)

The Eating Disorder Assessment for DSM-5 (EDA-5) is a semi-structured interview designed to assist in the diagnosis of feeding and eating disorders based on DSM-5 criteria (Tournayre et al., 2024). The EDA-5 was used to provide a structured assessment of eating disorder psychopathology and diagnostic status. EDA-5 demonstrates strong psychometric properties, with a mean test–retest reliability of *κ* = 0.87 (Tournayre et al., 2024).

#### 2.4.5 Demographic and background information survey

A Likert-type demographic and background information survey was administered to assess participants’ demographic and relevant background characteristics.

#### 2.4.6 EEG and event-related potential paradigm

An event-related potential (ERP) paradigm was applied to investigate participants’ neurophysiological responses to the experimental stimuli. EEG responses were recorded using a 128-channel BioSemi system at a sampling rate of 2,048 Hz with 24-bit resolution. Whole-brain analyses were conducted to localize participants’ responses during early and late time windows reflecting bottom-up and top-down processes.

#### 2.4.7 Palatability ratings

Following each food-related trial (120 trials in total), participants evaluated food palatability by responding to the question “Was the presented food palatable?” (translated from Greek). Although this question was adapted from the paradigm established by Frausto et al. (2022), the present study employed a binary yes/no response as defined *a priori* in our pre-registered protocol rather than a continuous rating scale. Binary classification paradigms are well established in food-cue research (Dondzilo et al., 2024; Ralph-Nearman et al., 2019). Additionally, the use of a binary yes/no response was chosen because it simplifies the participants’ decision to a categorical judgment (i.e., “palatable” vs. “not palatable”).

### 2.5 Stimuli

The food stimuli used for the purposes of the study were taken from the CROss-CUltural Food Image Database (CROCUFID) (Toet et al., 2019), which is an open-access dataset that contains 840 images consisting of 675 food images and 165 images of non-food items. The CROCUFID database includes images of sweet, savory, natural, and processed foods that are grouped into four categories: universal, Western, Asian, and unappealing. CROCUFID used a high-resolution standardized photographing protocol. Additionally, a standardization procedure was applied for image characteristics that are known to influence affective observer responses (e.g., texture, regularity, complexity, and colorfulness).

Prior to the food stimulus selection process, a clinical nutritionist assessed the pictures regarding their caloric content (per serving of 100 g) as in the study of Frausto et al. (2022). Food stimuli were categorized based on caloric content: HCF images contained ≥ 250 kcal, and LCF images contained ≤ 70 kcal. Food pictures that were difficult to identify or not representative of Western food culture were excluded. All selected HCF and LCF pictures were matched for the area occupied by the food item (in percentage of the total picture). Thirty HCF and 30 LCF pictures characterized as “fresh food” were chosen solely from the “Universal” and “Western” food categories to ensure cultural and dietary familiarity for the study sample. Each picture was presented twice. Additionally, color analyses were conducted indicating that main colors (blue, green, red, and yellow) did not differ between food categories (see Supplementary Table S1). The food pictures consisted of savory, sweet, natural, and transformed foods. The selected pictures were then used to create single-item food stimuli depicting either an HCF or an LCF presented on a white plate (1920 × 1,280).

HCF and LCF stimuli were selected to probe differential valuation of caloric content, consistent with prior work highlighting altered motivational responses to caloric density in AN patients (Cowdrey et al., 2013; Paslakis et al., 2016). Following the presentation of each food stimulus, participants had to answer a binary yes/no response question, which assessed their judgement about the palatability of the presented food stimuli.

The emotionally charged stimuli were taken from the International Affective Picture System (IAPS) dataset and were separated into positive and negative stimuli according to their valence and arousal properties (Lang et al., 1988). The positive stimuli comprised 30 pictures high in valence (*M* = 7.43, *SD* = 1.48) and low in arousal (*M* = 3.73, *SD* = 2.28), while the negative stimuli comprised 30 pictures low in valence (*M* = 2.56, *SD* = 1.62) and high in arousal (*M* = 6.67, *SD* = 2.11). Each picture was presented twice, with no behavioral responses required. The IAPS block was included to assess whether observed neural alterations were specific to food stimuli or reflected generalized affective processing differences, consistent with prior studies examining domain-specific versus general emotion-processing deficits in AN (Blechert et al., 2011a; Horndasch et al., 2018b).

### 2.6 Data analyses

Analyses of self-report questionnaires and behavioral measures were conducted in IBM SPSS Statistics (Version 27). Assumptions of normality and homogeneity of variance were evaluated, and when these assumptions were violated, nonparametric alternatives were used. Descriptive and inferential analyses were performed, with a significance level set at *p* < 0.05. Post-hoc analyses were conducted to further explore significant effects where appropriate.

The EEG preprocessing was conducted using the Brainstorm toolbox in MATLAB (Tadel et al., 2011), which is well documented and freely available for download online under the General Public License (GNU)^1^. Initially, the data were visually inspected, and bad channels were interpolated. EEG data were re-referenced to the common average. Artifacts generated by blinks or eye movements were corrected using the Signal-Space Projection (SSP) method via Brainstorm. Continuous data were separated into epochs of 800 ms, including 200 ms of a pre-stimulus interval. Epochs were baseline corrected using the interval from −100 to 0 ms. Data were filtered offline with a 0.5 Hz high-pass forward filter, a 30 Hz low-pass zero-phase filter, and an additional 50 Hz notch filter. The averages were calculated separately for each condition. Subsequently, the Global Field Power (GFP) was extracted from the averaged responses across all trials for each subject and each condition. Sensor-space nonparametric permutation paired t tests analysis was conducted (Monte Carlo method, 1,000 permutations) across all electrodes for the full 0–800 ms post-stimulus time window to compare the conditions (e.g., HCF vs. LCF, positive IAPS vs. negative IAPS). A minimum cluster duration of 20 ms was applied, and significance was assessed using a False Discovery Rate (FDR) correction (*α* = 0.05). These sensor-level analyses identified electrodes and time windows showing significant activity differences, providing information about the temporal dynamics and scalp topographies of neural responses. Significant electrode clusters and time windows guided subsequent source-level analyses, ensuring that source reconstruction focused on the periods and regions showing the most robust condition and group-related effects.

Current Density Reconstructions (CDRs) were calculated on the neural responses of each stimulus category and for each subject. A realistic approximation of the standardized boundary element model was used as a head model to solve the forward problem, based on the Colin27 template. The images were then smoothed through an isotropic Gaussian kernel of 10 mm. CDRs were calculated using Standardized Low Resolution Electromagnetic Tomography (sLORETA) for estimating source activity in the significant time windows to solve the inverse problem. The CDRs were estimated for the full duration of stimulus processing, covering both early and late processing stages. These were defined based on the identification of significant sensor-level data comparisons. CDRs were co-registered into the Montreal Neurological Institute (MNI) space using Statistical Parametric Mapping 12 (SPM12). For each time window the following analysis was performed on the corresponding CDRs.

Data were exported in NIfTI format and significant differences in cortical activity strength between groups (AN/AAN patients, HCs, OW/OB participants) and within-subjects factor condition (e.g., LCF pictures, HCF pictures, etc.) were identified, modeling the above-mentioned design via the Sandwich Estimator Toolbox for Longitudinal and Repeated Measures Data running on MATLAB. Specifically, a 3 × 2 mixed model analysis was implemented, with between-subjects factor Group and within-subjects factor condition. The predefined contrasts estimated via this analysis evaluated: (1) the main effect of Condition, testing whether the cortical sources activated during processing of each category differ independently of group; (2) the main effect of Group independently of the Conditions (whether the cortical sources activated in the processing in each group as a response to the stimuli category, independently of the type or weight category); and (3) the Group × Condition interaction effect, by testing group differences in specific condition contrasts (e.g., LCF vs. HCF processing) using two-sided contrasts derived from the 3 × 2 Group × Condition interaction. A wild bootstrap was used to estimate nonparametric inferences for the defined contrasts using 10,000 permutations, while the threshold-free cluster enhancement approach was used to calculate Family-Wise Error (FWE) corrected *p*-values at a voxel-cluster level, effectively controlling for multiple comparisons independently for each contrast. The reporting threshold was *p* < 0.05 corrected for multiple comparisons. This method ensured proper control of FWE for each contrast while simultaneously providing greater sensitivity than other methods across a wide range of test signal shapes and signal-to-noise ratio values. Subsequently, the time course of each voxel’s activity was extracted.

## 3 Results

All the participants of the current study were female (*N* = 51). Sex was defined as sex assigned at birth; gender identity was not assessed. Only female participants were included due to the higher prevalence of AN (Bulik et al., 2006) and as a methodological decision to reduce variance related to known sex differences in EEG responses (Cave and Barry, 2021).

Forty-eight participants (94.1%) were of Cypriot nationality and 3 (5.9%) were of Greek nationality. Within the AN/AAN group, 14 participants (82%) reported having experienced an eating disorder within the past 6 months, and 9 (52.9%) sought professional help. Regarding psychiatric comorbidities, within the AN/AAN group, 5 participants (29.4%) reported anxiety disorders, 3 (17.6%) reported depression, and 2 (11.8%) reported post-traumatic stress disorder, whereas borderline personality disorder and obsessive-compulsive disorder were each reported by 1 participant (5.9%). Comorbidity categories were not mutually exclusive, as some participants had more than one comorbidity. Furthermore, 2 OW/OB participants (11.8%) and 7 AN/AAN participants (41.2%) reported current medication use. Within the OW/OB group, 1 participant (5.9%) reported thyroid medication, and 1 (5.9%) reported insulin use. Within the AN/AAN group, 4 participants (23.5%) reported psychotropic medication use, 2 (11.8%) reported thyroid medication, and 1 (5.9%) reported insulin use.

All self-report measures violated the assumption of normality (Shapiro–Wilk tests, *p* < 0.05); therefore, group differences were examined using nonparametric Kruskal–Wallis H tests, with *post hoc* pairwise comparisons using Mann–Whitney *U* tests. Descriptive statistics and inferential results are presented in Table 1.

**Table 1:** Participant demographic and clinical characteristics across groups.

Variables	AN/AAN (*n* = 17)	Groups OW/OB (*n* = 17)	HCs (*n* = 17)	Statistic (*H, p*)	Post-hoc
Demographic characteristics					
Age (years)	24.6 (6.8)	30.65 (8.7)	22.6 (3)	*H*(2) = 14.61, *p* = 0.001	OW/OB > AN/AAN, HCs
BMI	20.7 (3.7)	30.2 (4.7)	21.6 (1.59)	*H*(2) = 30.96, *p* < 0.001	OW/OB > HCs > AN/AAN
Clinical characteristics					
Depression	18.4 (11.2)	8.12 (7.6)	6.59 (7.1)	*H*(2) = 14.66, *p* = 0.001	AN/AAN > HCs, OW/OB
Anxiety	19.2 (7.2)	5.5 (6.6)	7 (7.9)	*H*(2) = 22.40, *p* < 0.001	AN/AAN > HCs, OW/OB
Stress	22 (8.7)	10.9 (9.4)	11.8 (7.1)	*H*(2) = 14.50, *p* = 0.001	AN/AAN > HCs, OW/OB
EAT-26 total score	40.6 (16.5)	9.7 (5.7)	5.59 (4.8)	*H*(2) = 31.54, *p* < 0.001	AN/AAN > HCs < OW/OB
BI-AAQ	60.8 (22)	38.8 (16.7)	24 (11.8)	*H*(2) = 19.89, *p* < 0.001	AN/AAN > HCs < OW/OB

Values are presented as mean (SD). Group differences were assessed using Kruskal–Wallis H tests due to violations of normality. Significant effects were followed up using Post Hoc Mann–Whitney U tests. AN/AAN, Anorexia Nervosa/Atypical Anorexia Nervosa; OW/OB, Overweight/Obese; HCs, healthy controls.

**Figure 1:**
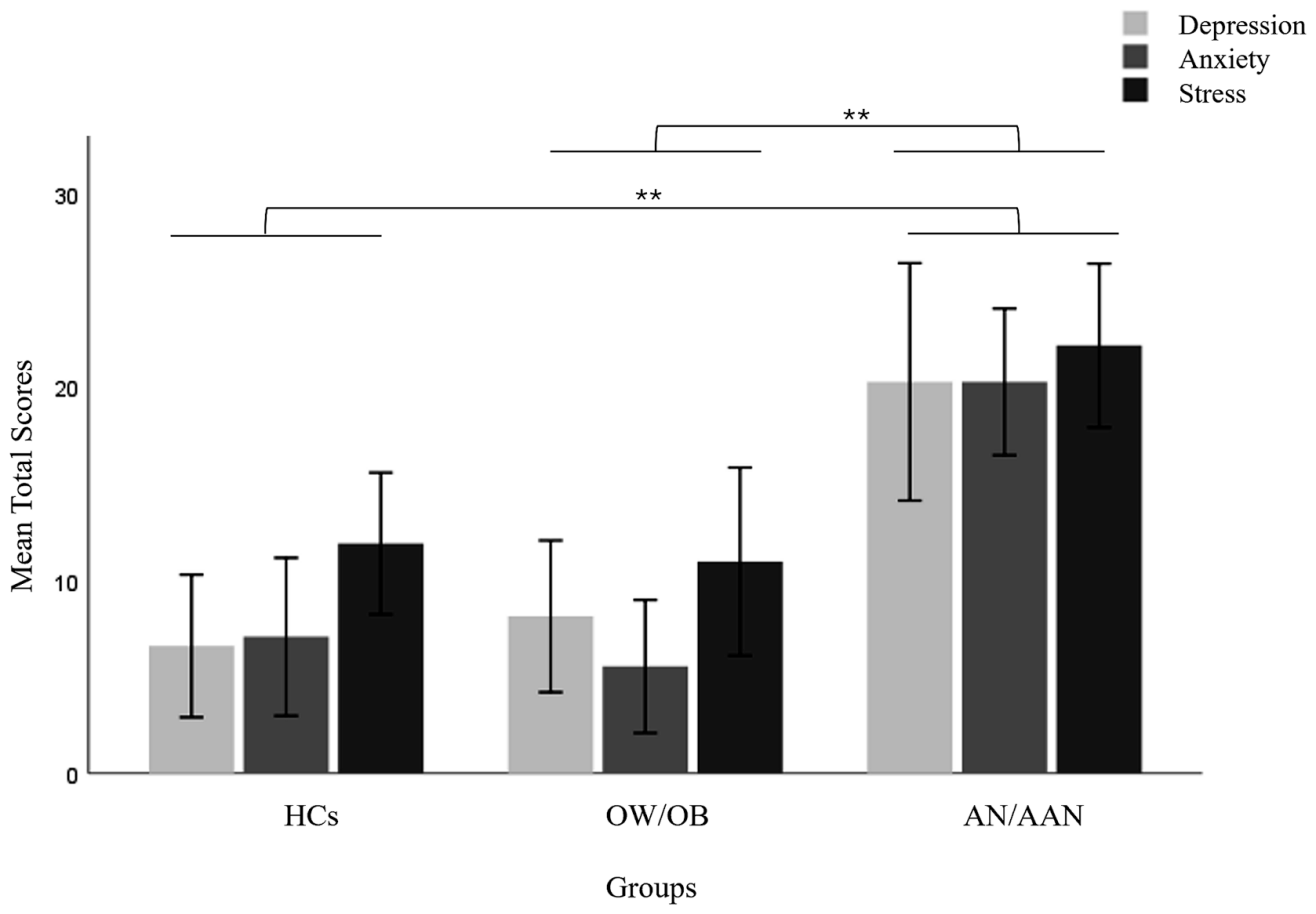
Mean DASS-21 scores for Depression, Anxiety, and Stress by group. Bars represent group means; Error bars represent 95% confidence intervals (*CI*) of the mean. HCs, Healthy Controls; OW/OB, Overweight/Obese; AN/AAN, Anorexia Nervosa/Atypical Anorexia Nervosa.

Significant group effects were observed across all three DASS-21 subscales (see Table 1 and Figure 1), with post hoc Mann–Whitney *U* tests indicating that the AN/AAN group had significantly higher levels of depressive, anxiety, and stress symptoms than the HCs (*p* < 0.01) and OW/OB participants (*p* < 0.01). No significant differences were observed between HCs and OW/OB participants on any DASS-21 subscale (*p* > 0.05). On the BI-AAQ, scores differed significantly across groups (see Table 1), with post hoc Mann–Whitney U tests indicating that the AN/AAN group exhibited significantly greater body-image inflexibility than HCs (*p* < 0.01) and OW/OB participants (*p* < 0.01). Additionally, OW/OB participants exhibited significantly higher body-image inflexibility compared to HCs (*p* < 0.05). Group differences were also observed for EAT-26 total scores, with post hoc comparisons showing that the AN/AAN group had significantly higher eating disorder-related pathology than HCs and OW/OB participants (*p* < 0.01), while OW/OB participants also scored significantly higher than HCs (*p* < 0.05).

### 3.1 Baseline characteristics across groups

Associations between group and baseline hunger, time since last caffeine consumption, and time since last nicotine use (see Table 2) were examined using Fisher’s exact tests, as the assumptions of the Pearson chi-square test were violated (i.e., more than 20% of cells had expected counts below 5). No significant group differences were observed for hunger (*p* = 0.62), caffeine consumption (*p* = 0.47), and nicotine use (*p* = 0.37).

**Table 2:** Baseline hunger and substances use variables by group.

Variables	AN/AAN (*n* = 17)	Groups OW/OB (*n* = 17)	HCs (*n* = 17)
Hunger prior to experiment			
Not hungry	9 (52.9%)	9 (52.9%)	8 (47.1%)
Slightly hungry	6 (35.3%)	7 (41.2%)	4 (23.5%)
Moderately hungry	2 (11.8%)	1 (5.9%)	4 (23.5%)
Very hungry	—	—	1 (5.9%)
Time since last caffeine consumption			
No caffeine consumed	8 (47.1%)	6 (35.3%)	11 (64.7%)
1 h	1 (5.9%)	—	—
2 h	—	—	—
3 h	—	2 (11.8%)	—
4 h	2 (11.8%)	4 (23.5%)	2 (11.8%)
≥ 5 h	6 (35.3%)	5 (29.4%)	4 (23.5%)
Time since last nicotine use			
No nicotine consumed	14 (82.4%)	17 (100%)	15 (88.2%)
1 h	—	—	—
2 h	—	—	1 (5.9%)
3 h	—	—	—
4 h	2 (11.8%)	—	1 (5.9%)
≥ 5 h	1 (5.9%)	—	—

Values are presented as counts, with percentages in parentheses. AN/AAN, Anorexia/Atypical Anorexia Nervosa; HCs, healthy controls; OW/OB, overweight/obese.

### 3.2 Palatability ratings

To examine group differences in palatability ratings, we conducted a mixed-design repeated-measures General Linear Model (GLM) with Food Type (LCF vs. HCF) as a within-subjects factor and Group (HCs, OW/OB, AN/AAN) as a between-subjects factor. The dependent variable was the total number of positive palatability responses (“Yes”) for LCF and HCF images. Assumption checks using Levene’s test indicated violations of homogeneity of variances for LCF stimuli, *F*(2, 48) = 5.26, *p* < 0.01, and HCF stimuli, *F*(2, 48) = 4.07, *p* < 0.05. Shapiro–Wilk W tests revealed non-normality for LCF stimuli in the OW/OB group, *W*(17) = 0.88, *p* = 0.04 and AN/AAN group, *W(17)* = 0.83, *p* < 0.01, whereas HCF stimuli were normally distributed (*p* > 0.05). Despite these violations, we proceeded with the GLM because it is generally more robust to modest deviations from normality and homogeneity when groups are balanced and sample sizes are equal (Knief and Forstmeier, 2021). The analysis revealed no significant difference in positive responses to LCF versus HCF stimuli, *F*(1, 48) = 1.37, *p* = 0.24, *η*^2^ = 0.02. Furthermore, there was no significant main effect of Group, *F*(2, 48) = 1.64, *p* = 0.20, *η*^2^ = 0.06 and no Food Type × Group interaction, *F*(2, 48) = 1.87, *p* = 0.16, *η*^2^ = 0.07, indicating that the palatability responses did not differ significantly across HCs, OW/OB, and AN/AAN participants (see Table 3 and Supplementary Figure S1).

**Table 3:** Palatability ratings for HCF and LCF images by group.

Food type	Group	M	SD	95% CI
LCF	Overall	41.39	10.65	[38.40, 44.39]
	HCs	42.76	4.97	[40.21, 45.32]
	OW/OB	40.29	10.65	[34.82, 45.77]
	AN/AAN	41.12	14.60	[33.61, 48.63]
HCF	Overall	39.18	12.84	[35.57, 42.79]
	HCs	43.94	8.14	[39.75, 48.13]
	OW/OB	39.76	9.99	[34.63, 44.90]
	AN/AAN	33.82	17.14	[25.01, 42.64]

Values represent the mean number of positive palatability (“Yes”) responses out of 60 trials for each food category. Sample size was identical across all cells (*N* = 17 per group). AN/AAN, Anorexia/Atypical Anorexia Nervosa; HCs, healthy controls; OW/OB, Overweight/Obese; HCF, High calorie food; LCF, low calorie food; M, Mean; SD, standard deviation, CI, confidence intervals.

As a *post hoc* exploratory analysis, following the approach of Cowdrey et al. (2013), patients with the binge-eating/purging subtype (AN-BP; *n* = 2) and AAN (*n* = 9) participants were excluded, leaving six Anorexia Nervosa-Restricting subtype (AN-R) participants in the experimental group. This exploratory analysis yielded a significant Food Type × Group interaction, *F*(2, 37) = 3.73, *p* = 0.03, *η_p_*^2^ = 0.17. Follow-up Bonferroni-corrected comparisons suggested that the observed interaction reflected lower positive palatability responses among AN-R participants to HCF (*M* = 29.83, *SE* = 5.05) than to LCF (*M* = 45.50, *SE* = 3.49), *F*(1, 37) = 8.27, *p* = 0.007, *η_p_*^2^ = 0.18, whereas the HCs (*p* = 0.71) and OW/OB groups (*p* = 0.87) showed no significant differences in their ratings between food types. Nonetheless, given the small number of AN-R participants, these findings should be considered preliminary.

### 3.3 Neurophysiological results

**Figure 2:**
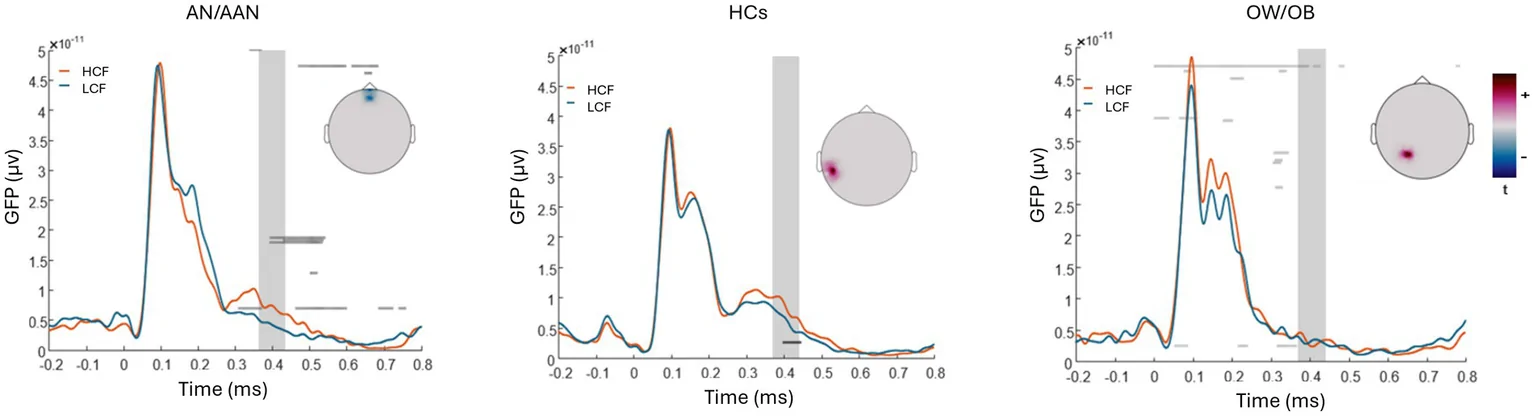
Within-group sensor-level EEG responses to HCF and LCF stimuli in AN/AAN, HCs, and OW/OB groups. Line plots show Global Field Power (GFP, μV) over time for HCF and LCF conditions. Shaded gray areas indicate statistically significant time windows. Scalp topographies illustrate the distribution of sensor-level activity during the significant time window (388–418 ms). In the scalp plots, red color indicates significant positive *t*-value and blue significant negative *t*-value in the comparison HCF-LCF. AN/AAN, Anorexia/Atypical Anorexia Nervosa; HCs, Healthy Controls; OW/OB, Overweight/Obese; HCF, High Calorie Food; LCF, Low Calorie Food.

Sensor-level within-group analyses were conducted for each group to examine the neurophysiological processing of HCF and LCF food stimuli. Figure 2 shows the time course of GFP for HCF and LCF stimuli in the AN/AAN, HCs, and OW/OB groups. The y-axis represents GFP (μV), reflecting the overall strength of the EEG signal across the scalp, while the x-axis represents time in milliseconds. Statistically significant differences between HCF and LCF stimuli (*p* < 0.05, FWE corrected) were observed in the 388–418 ms time window at the following electrodes: C17 (AFz), C18, and C19 (FPz) for the AN/AAN group; A8 for the OW/OB group; and D25 for HCs. These effects were reflected in both GFP amplitude and scalp topographies. The statistically significant time window corresponds to the N400 component, which is typically associated with higher-level cognitive evaluation and top-down semantic processing. The scalp topographies in Figure 2 represent the mean sensor-level activation for the HCF and LCF conditions during the significant time window.

**Figure 3:**
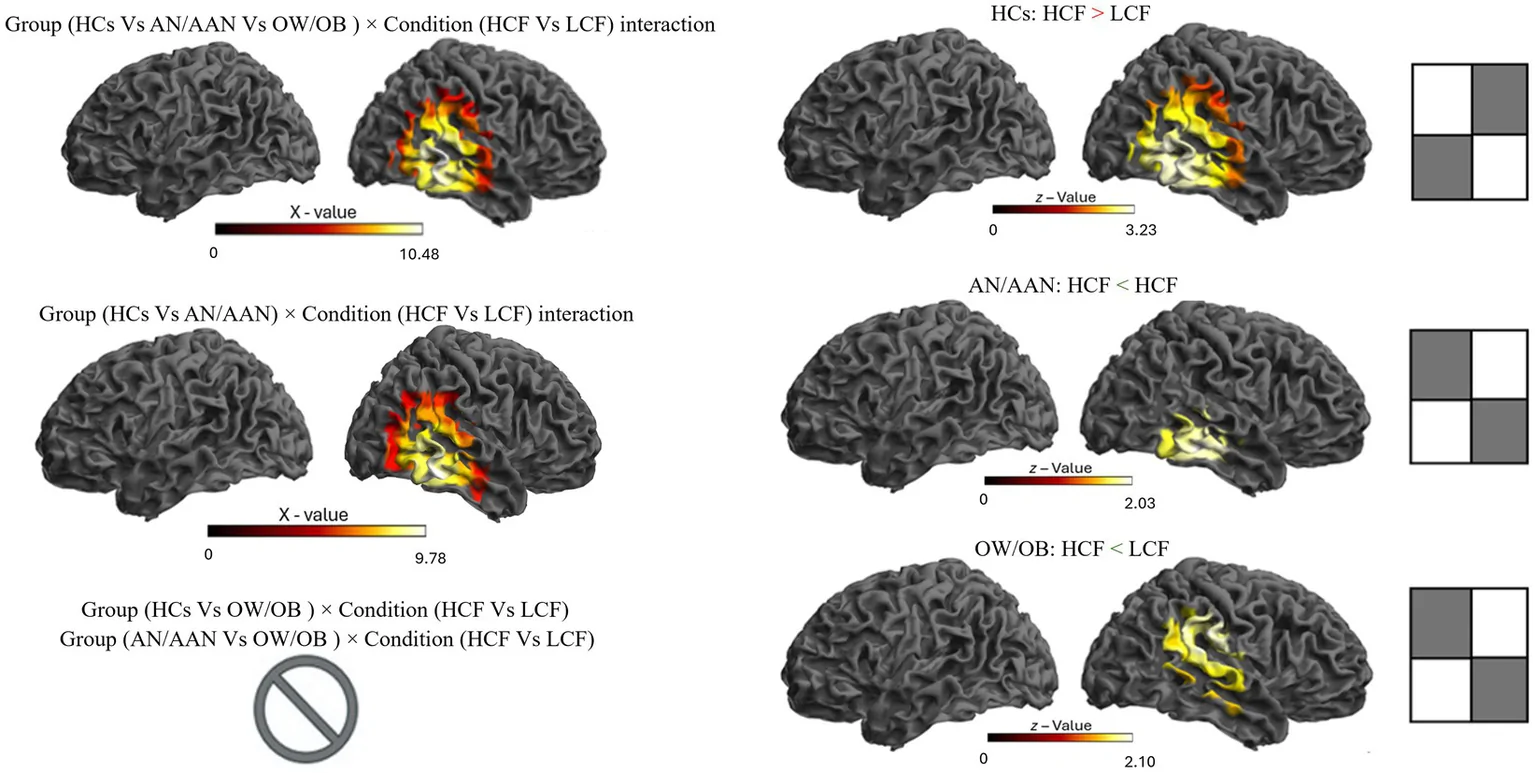
Source-level localization of group × condition interactions and main effects during the 388–418 ms time window. Brain regions showing significant group × condition interactions and main effects of condition (HCF *vs*. LCF). Interactions highlight differential responses across groups, while main effects indicate regions preferentially activated by high or low-calorie foods. AN/AAN, Anorexia/Atypical Anorexia Nervosa; HCs, Healthy Controls; OW/OB, Overweight/Obese; HCF, High Calorie Food; LCF, Low Calorie Food.

Subsequently, between-group source-level comparisons were conducted within the same statistically significant time window (388–418 ms) to examine the main and interaction effects of Group (AN/AAN, HCs, OW/OB) and Condition (HCF vs. LCF). All source-level analyses were performed using a Wild Bootstrap procedure with Family-Wise Error (FWE) correction. A statistically significant Group (HCs vs. AN/AAN vs. OW/OB) × Condition (HCF vs. LCF) interaction was identified (*p* = 0.03, cluster extent = 110 voxels) localized to the right fusiform gyrus extending laterally into the right medial and inferior temporal gyrus (peak MNI: 71, −43, −9; see Figure 3). Further analyses revealed that this interaction was primarily driven by the differences between HCs and AN/AAN participants. This was confirmed by a statistically significant, albeit modest, Group (HCs vs. AN/AAN) × Condition (HCF vs. LCF) interaction (*p*_FWE_ = 0.049, *F*(1, 32) = 11.64, cluster extent = 97 voxels) localized to the fusiform gyrus on the inferior surface of the right temporal lobe (peak MNI: 62, −43, −19). At the peak voxel, the unstandardized interaction effect size was *βˆ* = 1.16 × 10^−10^ [*SE* = 3.41 × 10^−11^, 95% CI (4.68 × 10^−11^, 1.86 × 10^−10^)], corresponding to a partial eta-squared of η_p_^2^ = 0.267. A voxel-wise spatial overlap analysis revealed high anatomical concordance between the within-group main effects: 100% of the AN/AAN cluster (HCF > LCF, 46 voxels) was physically nested within the larger HCs cluster (HCF < LCF, Dice Similarity Coefficient = 0.657). Deconstruction of the interaction within this shared locus confirmed a functional reversal in activation direction, with HCs exhibiting reduced activation for HCF relative to LCF (β_HC_ = −7.04 × 10^−11^), whereas the AN/AAN group exhibited increased activation for HCF relative to LCF (β_AN/AAN_ = +4.58 × 10^−11^). No statistically significant interaction effects (*p* > 0.05) were found when comparing the HCs and AN/AAN groups separately against the OW/OB group.

Concerning the main effects, during the same time window (388–418 ms), HCs exhibited greater activation in a right temporal cluster (*p* < 0.01, cluster extent = 98 voxels) encompassing the inferior temporal gyrus (peak MNI: 62, −52, −9) and extending into the middle temporal cortex, when processing HCF images. By contrast, in the AN/AAN group, a similar activation pattern was observed (peak MNI: 71, −33, −19) during the processing of LCF images (*p* < 0.05, cluster extent = 32 voxels), revealing an opposite direction of HCF–LCF neural responses relative to HCs. Similarly, the OW/OB group showed greater activation during LCF processing (*p* < 0.01, cluster extent = 35 voxels); however, this activation was localized to the supramarginal gyrus (peak MNI: 71, −32, 31) (see Figure 3).

**Figure 4:**
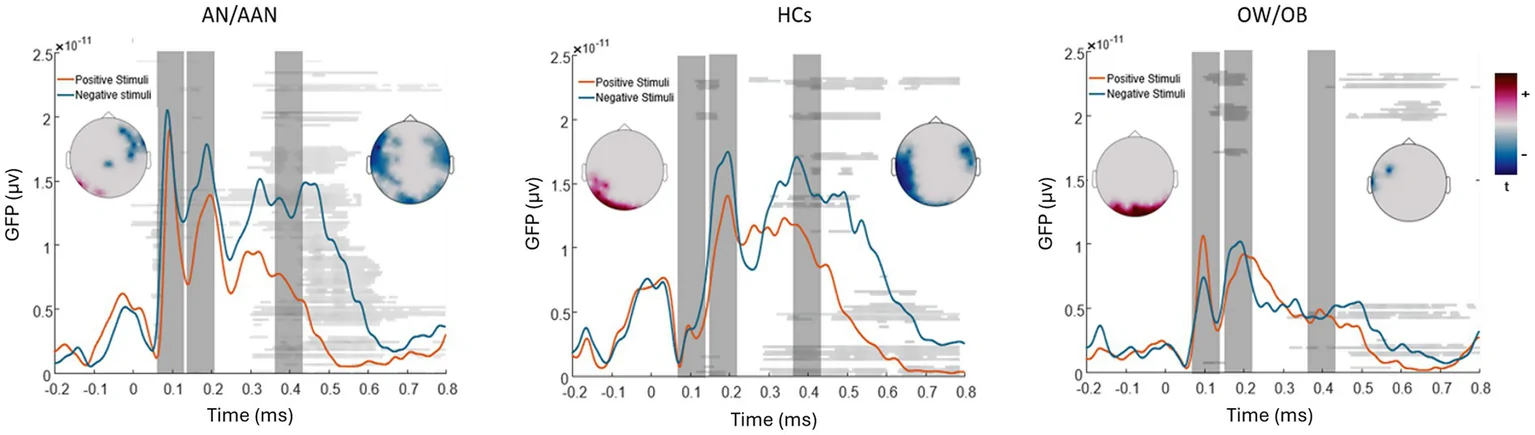
Within-group EEG responses to positive and negative IAPS stimuli in AN/AAN, HCs, and OW/OB groups. Line plots show Global Field Power (GFP, μV) over time for positive and negative conditions. Shaded gray areas indicate statistically significant time windows (82–121 ms, 175–220 ms, and 388–418 ms). Scalp topographies depict sensor-level activity during the significant time windows. In the scalp plots red color indicates significant positive t-value and blue significant negative *t*-value in the comparison positive–negative IAPS. AN/AAN, Anorexia/Atypical Anorexia Nervosa; HCs, Healthy Controls; OW/OB, Overweight/Obese.

Furthermore, participants’ responses to emotionally charged IAPS stimuli were analyzed to assess whether AN/AAN participants process these images similarly to how they process HCF and LCF stimuli. Within-group sensor-level analyses revealed three statistically significant (*p* < 0.05) time windows: 82–121 ms, 175–220 ms, and 388–418 ms, with the latter corresponding to the same time window that was significant during food stimulus processing (see Figure 4).

Although sensor-level analyses indicated significant effects (*p* < 0.05) for all the above-mentioned time windows, FWE-corrected source-level analyses showed no statistically significant interaction effects between the three groups (*p* > 0.05).

**Figure 5:**
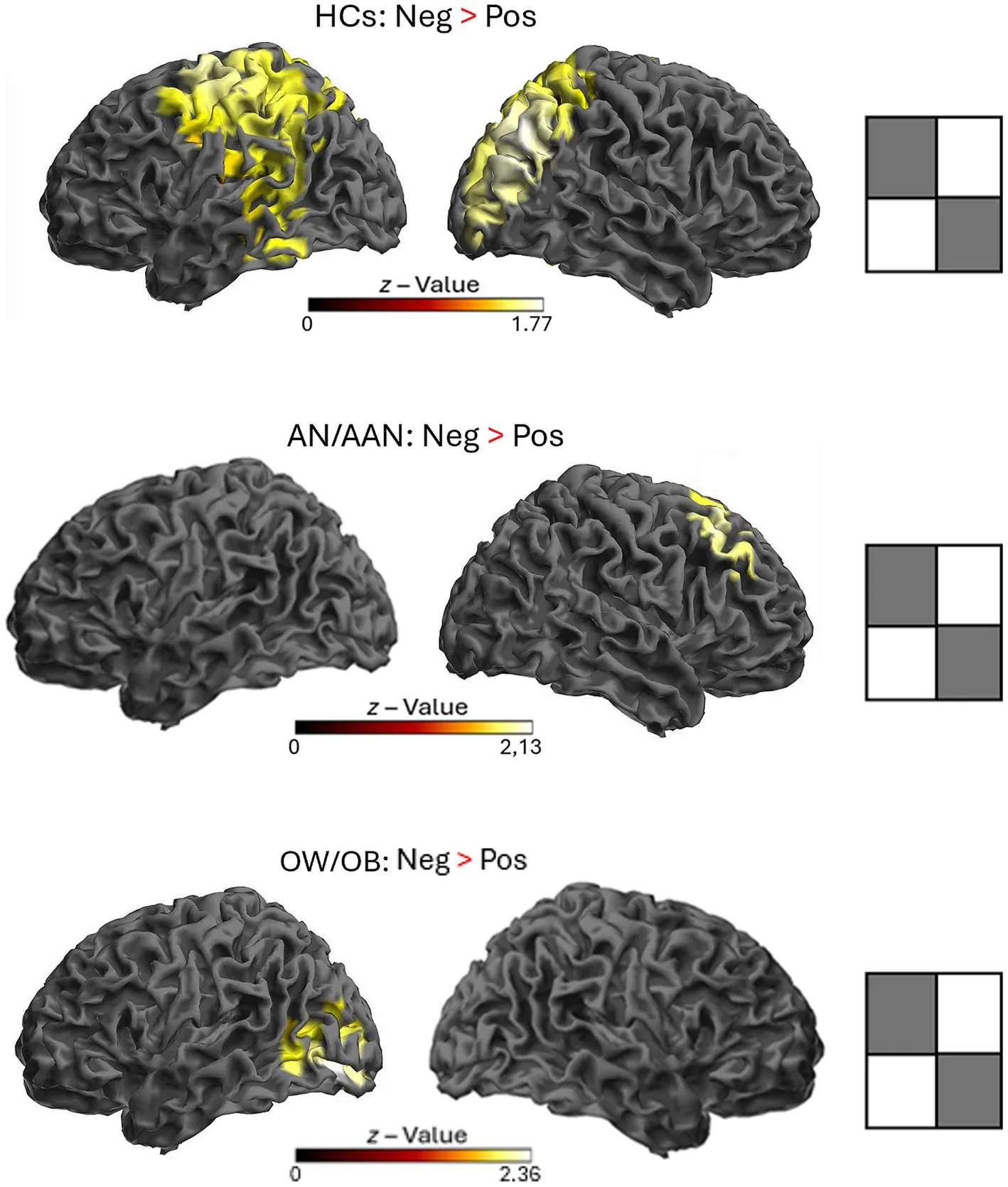
Source-level uncorrected main effects of emotional valence during IAPS processing (388–418 ms) in AN/AAN patients, OW/OB participants, and HCs. Brain regions showing significant main effects of condition (positive vs. negative stimuli) are displayed. AN/AAN, Anorexia Nervosa/Atypical Anorexia Nervosa; HCs, Healthy Controls; OW/OB, Overweight/Obese; Neg, Negative; Pos, Positive.

Given its relevance as the main time window in the food-stimulus analyses, the 388–418 ms interval was further examined in exploratory source-level analyses using an uncorrected threshold (Wild Bootstrap; *p* < 0.05). The results showed significant main effects of Condition (Positive vs. Negative IAPS) across all three groups with greater brain activation observed for negative IAPS, as illustrated in Figure 5. In the AN/AAN group, the effects appeared in the right superior frontal lobe (peak MNI: 23, 26, 71; cluster extent = 31 voxels). In the HCs, the uncorrected effects were located in the right angular gyrus extending into the lateral occipital cortex (peak MNI: 52, −71, 30; cluster extent = 296 voxels) as well as in the left precentral gyrus (peak MNI: −35, −12, 70), and the left Supplementary Motor Area (peak MNI: −6, −13, 60; cluster extent = 296 voxels). For the OW/OB group, the main effects were observed in the left inferior occipital cortex (peak MNI: −35, −81, −10; cluster extent = 32 voxels).

## 4 Discussion

The present study aimed to examine EEG responses to HCF and LCF stimuli among AN/AAN patients, OW/OB participants, and HCs, investigate their relationship with palatability ratings, and determine whether altered neural activation during food processing was largely food-specific or extended to general emotional processing (IAPS). Results showed a dissociation between the relatively preserved explicit hedonic evaluations (palatability ratings) of AN/AAN and their altered higher-order neural processing of food. Our EEG findings showed a Group × Condition interaction in AN/AAN participants within the N400 time window, localized to the right inferior temporal region and indicating altered late semantic/evaluative processing of food cues. Importantly, this neural alteration did not generalize to emotionally salient IAPS stimuli, supporting the interpretation that the identified semantic disturbance was largely food-specific. Together, these findings support the view that food-processing abnormalities in AN/AAN arise from disrupted late semantic/evaluative mechanisms rather than an impaired capacity to experience the hedonic value of foods. However, clinical heterogeneity should be considered when interpreting the present findings. Prior neuroimaging research using food-related paradigms in AN has highlighted the importance of examining AN both as a broader clinical group and through subtype-specific analyses, as these approaches may reveal distinct patterns of neural dysfunction (Brooks et al., 2012). Accordingly, the observed findings in the present AN/AAN sample may not be uniformly shared across AN-R, AN-BP, and AAN, and the relative contribution of each clinical presentation to the observed group-level effect remains unclear.

Our self-report findings, in line with prior work, indicate that AN/AAN patients exhibit elevated psychological distress (stress, depression, and anxiety) and eating disorder psychopathology compared to HCs (Özcan and Çevreli, 2025; Sternheim et al., 2015). Additionally, AN/AAN patients exhibit the highest body image inflexibility, followed by OW/OB participants, who in turn show greater inflexibility than HCs. These findings align with prior evidence, suggesting that body image inflexibility may represent an important contributor to disordered eating (Zou et al., 2022).

Our behavioral findings suggest that the maladaptive food choice behaviors observed in AN/AAN are unlikely to be driven by a disturbed capacity to experience the hedonic/affective properties of foods. Rather, these patterns point to an altered valuation hierarchy. Food choice differentiation in AN/AAN may be primarily driven by abnormal food-related motivational processes, particularly alterations in the incentive salience (“wanting”) rather than explicit palatability ratings (“liking”) (Cowdrey et al., 2013). This is also supported by previous studies reporting that AN patients’ food choices are more strongly influenced by perceived healthiness relative to tastiness compared with HCs (Schröder et al., 2025; Steinglass et al., 2015). Nonetheless, by following the exploratory approach of Cowdrey et al. (2013), we have isolated the AN-R subgroup (*n* = 6) and observed lower palatability ratings for HCF than LCF stimuli relative to the HCs and OW/OB participants. Although preliminary, our exploratory findings are broadly consistent with the interpretation of Cowdrey et al. (2013) raising the possibility that individuals with purely restrictive eating patterns (AN-R) may rely, at least in part, on explicit top-down strategies (e.g., deliberate cognitive reappraisal or explicit devaluation strategies) that may contribute to the suppression of normative appetitive drives and the maintenance of restrictive control. Nonetheless, our behavioral findings indicate that overall food choice differentiation in AN/AAN is not primarily driven by altered hedonic responses, but rather by higher-order cognitive and motivational mechanisms.

Concerning the EEG results, our findings support the hypothesis that AN/AAN patients exhibit altered neural processing of food stimuli compared with HCs and OW/OB participants (see Figure 3). Specifically, a reversed HCF–LCF neural response pattern was observed in AN/AAN compared with HCs, which was characterized by greater neural engagement during LCF processing compared with HCF processing. This prominent reversal pattern emerged as an exploratory finding rather than an *a priori* hypothesis and was observed within the N400 time window (388–418 ms). The N400 is associated with higher-level semantic and evaluative processing, including meaning integration and prediction/expectancy mechanisms (Federmeier, 2007; Kutas and Federmeier, 2011). Furthermore, the N400 is modulated by participants’ prior experiences and attentional allocation (Kutas and Federmeier, 2011). Moreover, in ERP studies, the N400 appears when target stimuli do not smoothly match the semantic features or expectations of the participants, causing greater demands on semantic processing and meaning integration (Blechert et al., 2011b; Lahtinen et al., 2019; Rojas et al., 2024). Image-based stimuli tend to elicit N400 effects with a more anterior topography (Kutas and Federmeier, 2011), which was also observed in our study. Pergola et al. (2017) further showed that N400 topography can vary across experimental manipulations and participant groups, with more anterior effects when pictures are used as stimuli.

Our source level analyses focusing on the N400 time window localized the Group × Food-condition interaction to the medial and inferior temporal cortex extending to the fusiform gyrus. This pattern suggests the engagement of a broader network supporting the integration of visual food representations with higher-order contextual or evaluative information, and it is consistent with the later timing of the observed N400 component. Specifically, in the AN/AAN group, these regions, and particularly the inferior temporal and fusiform areas, were more strongly engaged by LCF than by HCF stimuli.

The involvement of the temporal cortex observed in the present study aligns with prior evidence. Neuroimaging findings have demonstrated that the fusiform gyrus and inferior temporal areas are activated during visual processing of food stimuli (van der Laan et al., 2011; Adamson and Troiani, 2018). Moreover, structural abnormalities in AN patients have been reported in temporal regions, among other areas, including reduced gray matter volume (Amianto et al., 2013; Friederich et al., 2012), and white matter volume (Frank et al., 2013). MEG findings also point toward altered neural responses to food stimuli, independent of caloric value, in current and recovered AN patients across various regions, with the inferior temporal gyrus showing enhanced activity at 320 ms in recovered patients, supporting a preliminary interpretation of generally altered food-related neural processing (Godier et al., 2016). Together, these findings are consistent with the heightened engagement of fusiform and temporal regions observed in the AN/AAN group during the N400 stage in our study, suggesting a possible neural substrate for altered semantic processing of food stimuli. Additionally, prior fMRI studies using gustatory food stimuli as well as visual body stimuli have reported significant involvement of temporal regions in AN patients, particularly the medial and inferior temporal gyrus (Castellini et al., 2013; Vocks et al., 2011). Specifically, Castellini et al. (2013) reported increased inferior temporal gyrus activation in AN patients among other regions compared to HCs when viewing oversized images of their own bodies, while HCs showed a reversed pattern, with greater inferior temporal activation in the undersized condition. Converging with the above pattern, Vocks et al. (2011) proposed that increased medial temporal activation in AN patients during gustatory stimulation in a hunger state may reflect weight gain anxiety. These findings show that temporal involvement as a response to salient food, body, or weight related stimuli is a common feature in AN.

To reveal whether the processing disturbances in AN were primarily food-specific or reflected a more generalized disruption in emotional processing, we examined responses during the IAPS block within the N400 time window (388–418 ms). AN/AAN participants did not show the same neural pattern for emotionally charged IAPS images as they did for food stimuli, showing that the altered neural modulation observed during the processing of food images was largely food-specific rather than reflecting a broad emotional processing impairment. Additionally, during the IAPS block, all groups demonstrated greater activation to negative pictures (uncorrected for multiple comparisons), which are perceived as more emotionally intense than positive pictures (Bradley et al., 2003).

Taken together, the present findings provide novel evidence for altered late semantic processing (N400) in AN/AAN that appears to be largely food-specific and independent of their explicit hedonic experience. Notably, although not pre-specified as a directional hypothesis, a prominent reversed HCF–LCF neural response pattern emerged in AN/AAN relative to HCs, characterized by greater neural engagement during LCF than HCF processing. The enhanced N400 responses toward LCF stimuli observed in both AN/AAN and OW/OB participants, localized to inferior temporal cortex, potentially reflect a mismatch or incongruence between semantic threat-related food associations (e.g., fear of weight gain and loss of control) and semantic safety-related associations linked to LCF stimuli (e.g., safe, permitted, non-fattening foods). This mismatch appears to reflect stimulus-driven interactions with semantic memory networks. Our findings suggest that altered higher order semantic and valuation processes contribute to the evaluation of food-related stimuli in AN/AAN, with potential implications to food-related restrictive eating behaviors. Moreover, our exploratory finding that AN-R patients rated HCF as less palatable suggests a subtype-specific pattern of explicit devaluation of HCF stimuli. Future research should further investigate neurophysiological and behavioral differences across AN subtypes (AN-R vs. AN-BP) and AAN during food processing. Beyond hedonic experience (palatability), other motivational factors contributing to restrictive control, including craving (“wanting”) and the perceived healthiness of food stimuli should be investigated.

A limitation of the present study is the presence of demographic differences across groups. Specifically, the AN/AAN group was not homogeneous in terms of BMI, as it included both AN and AAN individuals, whereas the OW/OB group was older than the other two groups. Age-related differences may influence the latency of the N400 response (Kutas and Iragui, 1998). However, all participants were within the same developmental stage (young adulthood), reducing the likelihood of substantial age-related differences in neural processing. Furthermore, an analysis of N400 peak latency within the investigated time window revealed no systematic group differences. A one-way ANOVA showed no significant effect of group, *F*(2, 48) = 1.17, *p* = 0.318, and pairwise comparisons were likewise non-significant (HC vs. OW/OB: *t*(32) = −1.42, *p* = 0.164; HC vs. AN/AAN: *t*(32) = −0.21, *p* = 0.838; OW/OB vs. AN/AAN: *t*(32) = 1.17, *p* = 0.249). Potential psychiatric comorbidities and medication use, particularly in the AN/AAN group, may have also influenced our findings. Medication use and comorbidities were descriptively reported, however, their inclusion as covariates was not considered statistically appropriate given the small sample size and the heterogeneous and sparse distribution of medication and comorbidity profiles within the AN/AAN group. Consequently, the potential influence of these factors on the observed neurophysiological findings cannot be fully excluded. Previous neuroimaging evidence suggests that distinct patterns of neural activity emerge when AN subtypes are examined independently (Brooks et al., 2012); however, due to limited sample size, no separate neuroimaging analyses were conducted. Consequently, our findings cannot account for how clinical heterogeneity might differentially modulate calorie evaluation and food processing. Thus, the present findings cannot establish whether the observed behavioral and neural pattern is shared across these clinical presentations or is disproportionately influenced by a specific subtype or weight status. Another potential limitation is the participants’ anticipation of making a palatability-related judgment (yes/no) in the food-dedicated block, although excluded from the EEG analyses, might have still subtly influenced our findings. A further limitation concerns the presentation of food stimuli twice within each block, despite randomization and intervening trials, repetition or familiarity effects may have still influenced N400 responses. Variations in treatment stage and illness duration may also represent a limitation (Jáuregui-Lobera, 2011). Participants’ satiety was assessed using a self-report measure prior to the experiment; no objective measure of satiety or pre-session food intake was utilized. Moreover, deficits in interoceptive awareness in AN may have affected their ability to accurately detect and report internal hunger state (Fassino et al., 2004). Lastly, the generalizability of our findings is limited, as the sample consisted solely of female participants.

## 5 Conclusion

In summary, our neurophysiological results indicate that individuals with AN/AAN exhibit altered neural processing of food stimuli within the N400 time window. Although not hypothesized *a priori*, the altered neural processing observed was characterized by a prominent reversed HCF–LCF neural response pattern that emerged in AN/AAN relative to HCs. This neural pattern was localized to temporal regions, including the fusiform and inferior-medial cortices. The enhanced neural responses of AN/AAN and OW/OB groups compared to HCs toward HCF and LCF stimuli indicate an altered caloric-dependent semantic processing of food stimuli, highlighting a potential neural substrate through which food-related beliefs and semantic interpretations shape restrictive eating behavior. Importantly, this neural differentiation was largely food-specific, as responses to IAPS stimuli during the same time window did not elicit comparable activation patterns in the fusiform or inferior temporal regions. Moreover, the dissociation observed between objective (EEG) and subjective (food palatability ratings) approaches suggests that food processing abnormalities in AN/AAN may primarily reflect altered evaluative or semantic representations of food rather than impaired capacity to experience hedonic value per se. Nonetheless, exploratory behavioral findings suggested that explicit suppression of liking for HCF stimuli may appear selectively in the AN-R subtype (*n* = 6), pointing to an additional distinct cognitive mechanism.

## Data Availability

The datasets presented in this study can be found in online repositories. The names of the repository/repositories and accession number(s) can be found below: https://gin.g-node.org/Panagiotis/Anorexia-Nervosa-Food-and-IAPS-experiment.git.

## Footnotes

1
http://neuroimage.usc.edu/brainstorm
