# Supplementary material for: Altered late semantic processing of food stimuli in female anorexia nervosa and atypical anorexia nervosa patients
Source: Front Hum Neurosci. 2026 Sep 3;20:1912696. doi: 10.3389/fnhum.2026.1912696 (PMC13582553; doi:10.3389/fnhum.2026.1912696)
Supplement: Supplementary file 1 [file Table_1.docx]

**Appendix A. Supplementary data**

**Table S1.** Univariate analysis for color variables by group

| Variables | LC stimuli *M* (*SD*) HC stimuli *M* (*SD*) | | *F* | *p* |
| --- | --- | --- | --- | --- |
| Colorfulness | 47.8 (15.8)  .48 (.11) | 49.9 (10.9)  .49 (.05) | .37 | .54 |
| Red |  |  | .49 | .48 |
| Green | .33 (.08)  -.76 (.16) | .34 (.02)  -.83 (.13) | 1.04 | .31 |
| Blue (log) |  |  | 3.34 | .07 |

Note. *F*-values correspond to univariate analyses comparing groups (*df* = 1, 58; 60 pictures total). *P*-values indicate the significance of the group differences. Blue values were log-transformed prior to analysis to meet normality. Values represent the mean number (*M*) of positive (“Yes”) responses and standard deviation (*SD*) for each color variable. *Abbreviations*: LC, Low-Calorie; HC, High-Calorie; *M*, Mean; *SD*, Standard Deviation.
